# Response of Osteosarcoma Cell Metabolism to Platinum and Palladium Chelates as Potential New Drugs

**DOI:** 10.3390/molecules26164805

**Published:** 2021-08-08

**Authors:** Ana S. Martins, Ana L. M. Batista de Carvalho, Maria P. M. Marques, Ana M. Gil

**Affiliations:** 1 CICECO—Aveiro Institute of Materials (CICECO/UA), Department of Chemistry, University of Aveiro, Campus Universitário de Santiago, 3810-193 Aveiro, Portugal; ascm@ua.pt; 2 Unidade de I&D Química-Física Molecular, Department of Chemistry, University of Coimbra, Rua Larga, 300-535 Coimbra, Portugal; almbc@uc.pt; 3 Department of Life Sciences, Faculty of Science and Technology, University of Coimbra, Calçada Martim de Freitas, 3000-456 Coimbra, Portugal

**Keywords:** metal chelates, human osteosarcoma cells, palladium, platinum, spermine, NMR, metabolomics, endometabolome

## Abstract

This paper reports the first metabolomics study of the impact of new chelates Pt_2_Spm and Pd_2_Spm (Spm = Spermine) on human osteosarcoma cellular metabolism, compared to the conventional platinum drugs cisplatin and oxaliplatin, in order to investigate the effects of different metal centers and ligands. Nuclear Magnetic Resonance metabolomics was used to identify meaningful metabolite variations in polar cell extracts collected during exposure to each of the four chelates. Cisplatin and oxaliplatin induced similar metabolic fingerprints of changing metabolite levels (affecting many amino acids, organic acids, nucleotides, choline compounds and other compounds), thus suggesting similar mechanisms of action. For these platinum drugs, a consistent uptake of amino acids is noted, along with an increase in nucleotides and derivatives, namely involved in glycosylation pathways. The Spm chelates elicit a markedly distinct metabolic signature, where inverse features are observed particularly for amino acids and nucleotides. Furthermore, Pd_2_Spm prompts a weaker response from osteosarcoma cells as compared to its platinum analogue, which is interesting as the palladium chelate exhibits higher cytotoxicity. Putative suggestions are discussed as to the affected cellular pathways and the origins of the distinct responses. This work demonstrates the value of untargeted metabolomics in measuring the response of cancer cells to either conventional or potential new drugs, seeking further understanding (or possible markers) of drug performance at the molecular level.

## 1. Introduction

Cancer is a public health concern as the second leading cause of death worldwide [1]. In particular, osteosarcoma (OS) is an aggressive and common type of primary malignant bone tumor. Even though OS can develop at any age, children, teenagers and young adults (aged 10–30 years) are the most affected populations [2]. Therefore, as for other malignancies, there is a continuing need for effective anticancer agents associated to minimal adverse side effects. Since the discovery of cisplatin (cDDP) and its approval as a antineoplastic agent [3], this platinum Pt(II)-based drug has been successfully used in the treatment of several types of cancer, e.g., brain, breast, lung, ovarian and head cancers [4]. However, cDDP is associated to serious systemic toxicity (e.g., nephrotoxicity and hepatotoxicity) and acquired resistance. In order to overcome these deleterious side effects and unveil new potential antineoplastic agents, other Pt(II) anticancer drugs such as oxaliplatin (OXA) and carboplatin, have been developed [5], although still associated to several often serious side effects [6]. Due to the chemical similarity between Pd(II) and Pt(II) ions, palladium chelates [7] have drawn increasing interest in this context [8,9,10,11,12,13,14,15,16,17,18,19,20,21,22,23]. In spite of their favorable antiproliferative and cytotoxic activities [12,15,21,22,24], their high lability requires strongly coordinating ligands and reasonably nonlabile leaving groups to ensure stability. Therefore, biogenic polyamines such as spermine (Spm = H_2_N(CH_2_)_3_NH(CH_2_)_4_NH(CH_2_)_3_NH_2_) have been used as coordinating ligands, since they form stable polynuclear chelates with both Pt(II) and Pd(II) [25,26]. These polynuclear agents allow the formation of long-range inter- and intra-strand crosslinks within DNA, leading to severe DNA damage and to improve in vitro antitumor efficacy, namely towards human metastatic breast cancer [13,14,18,19,24], ovarian cancer [10], oral cancer [9] and OS [21]. Interestingly, Pd_2_Spm has been shown to be more effective than its Pt(II) analogue against cell lines of triple negative breast [9] and ovarian [10] cancers, as well as of osteosarcoma [23]. Regarding the latter, Pd_2_Spm was observed to correspond to a lower IC_50_ than Pt_2_Spm, cDDP or OXA [23].

Improved knowledge on the impact of Pt(II) and Pd(II) agents on cancer cell metabolism may contribute for a better understanding of their mechanisms of action at the molecular level and enable the identification of potential markers of therapy response. Untargeted Nuclear Magnetic Resonance (NMR) metabolomics (a strategy with large potential in clinical applications through biofluids and tissue analysis) allows for the rapid metabolic profiling of cultured cells, to characterize their metabolome and relate metabolic deviations to cellular response to therapy. Indeed, NMR metabolomics has been applied to evaluate the metabolic response to cDDP of lung [27,28], brain [29,30,31], breast [32,33,34], ovarian [35] cancer cell lines, as well as of OS [36,37], mostly by analyzing whole or lysed cells or, in a few studies, focusing on cell extracts and cell media [30,31,35]. As for other conventional Pt(II) drugs, the metabolic impact of OXA on polar extracts of an hepatocellular carcinoma cells has been evaluated by NMR metabolomics [38], as has that of carboplatin (in combination with other drugs) on the exometabolome of oral squamous carcinoma cells [39]. To the best of our knowledge, the impact of Pd_2_Spm on human OS (MG-63 cell line) has only been evaluated, by the authors themselves, through high resolution magic angle spinning (HRMAS) NMR metabolomics of lysed cells [21], having been shown to induce less metabolic changes than cDDP despite its comparatively higher cytotoxicity. To our knowledge, no reports are to be found on similar studies for the Pt(II) analogue, Pt_2_Spm.

**Figure 1:**
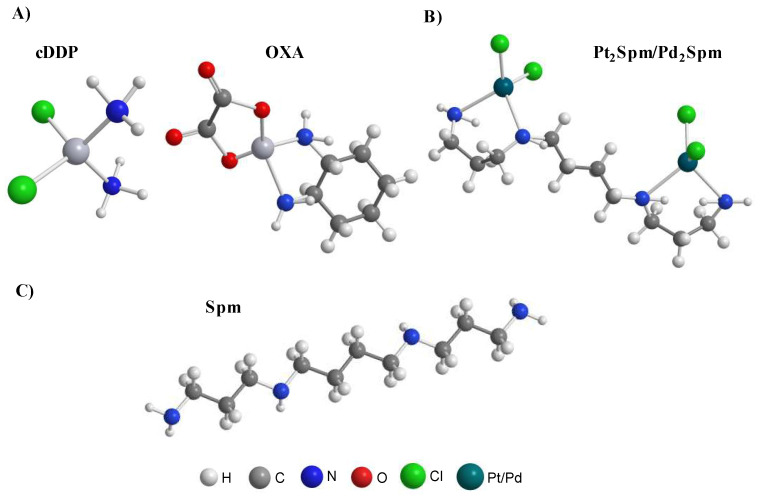
Schematic structural representations of: (**A**) conventional Pt(II) drugs cisplatin (cDDP) and oxaliplatin (OXA), (**B**) Pt_2_Spermine (Spm) and Pd_2_Spm and (**C**) the polydentate biogenic amine Spm.

In this work, a NMR metabolomics strategy was carried out, for the first time to our knowledge, to assess and compare the impacts of cDDP, OXA, Pt_2_Spm and Pd_2_Spm on the metabolism of human osteosarcoma MG-63 cells, aiming to contribute towards a better understanding of these drugs’ mechanisms of action and unveil metabolic markers of OS response to therapy. Figure 1 shows the structures of the two Pt(II) clinically used drugs cDDP and OXA, along with those of the Pt(II) and Pd(II) spermine chelates. While the metabolic profile of MG-63 cells exposed to cDDP and Pd_2_Spm has been previously investigated in lysed cells [21,36,37], as mentioned above, this work provides additional relevant information on these, since it addresses cell extracts (in particular, polar extracts). By combining multivariate and univariate statistical analysis of NMR time course data, valuable information is obtained on the dynamic metabolic response of osteosarcoma cells to both conventional and new Pt(II) and Pd(II) agents.

## 2. Results

**Figure 2:**
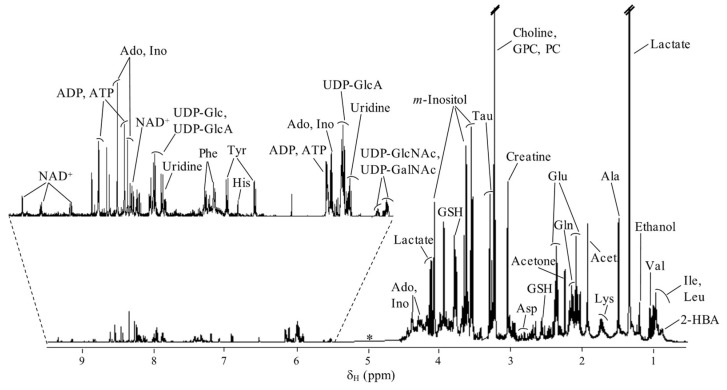
Average 500 MHz ^1^H NMR spectrum of polar extracts of control MG-63 cells for t = 0 h. Main assignments are indicated. ^✽^: Cut-off spectral region from multivariate analysis due to water suppression (δ 4.55–5.40). Metabolite abbreviations: three-letter code used for amino acids; 2-HBA: 2-hydroxybutyrate; Acet., acetate; Ado, adenosine; ADP, adenosine diphosphate; ATP, adenosine triphosphate; GPC, glycerophosphocholine; GSH: glutathione (reduced); Ino, inosine; NAD^+^: nicotinamide adenine dinucleotide; PC: phosphocholine; UDP-GalNAc: uridine 5′-diphospho-*N*-acetylgalactosamine; UDP-Glc: uridine 5′-diphosphosphate glucose; UDP-GlcA: uridine 5′-diphosphoglucuronic acid; UDP-GlcNAc: uridine 5′-diphospho-*N*-acetylglucosamine.

A typical ^1^H NMR spectrum of a polar extract (obtained by the methanol/chloroform/water method) of non-exposed MG-63 cells at t = 0 h (Figure 2) shows the predominance of amino acids, choline compounds (free choline, phosphocholine (PC), glycerophosphocholine (GPC), nucleotides and derivatives, reduced glutathione (GSH), lactate and *m*-inositol, building up on previous reports of perchloric acid MG-63 cell extracts [40,41]. All hereby assigned compounds may be found in Table S1 (Supplementary Materials). As expected, the cell extract spectrum shows significantly richer information regarding number and resolution of metabolite signals in all regions of the spectrum, compared to the previously reported ^1^H HRMAS NMR spectrum of the same cell line [21,36,37].

**Figure 3:**
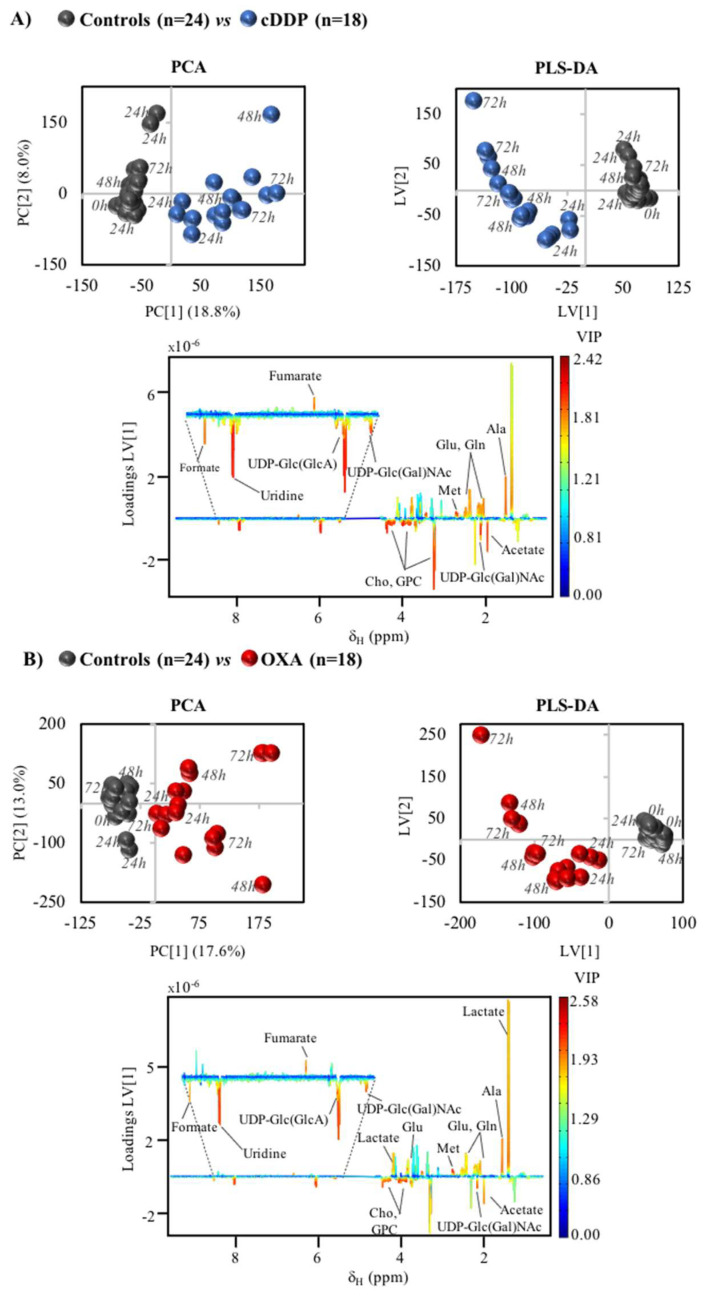
Multivariate analysis of MG-63 cells exposed to cDDP and OXA. (**A**) PCA (left) and PLS-DA (right, with LV = 2, R^2^_x_ = 0.259, R^2^_y_ = 0.977, Q^2^_cum_ = 0.933) scores plots obtained for ^1^H NMR spectra of cells exposed to 30 μM cDDP, along with LV1 loadings plot (bottom) colored by variable importance projection (VIP) and with main peak assignments; (**B**) PCA (left) and PLS-DA (right, with LV = 2, R^2^_x_ = 0.285, R^2^_y_ = 0.954, Q^2^_cum_ = 0.884) scores plots obtained for 100 µM OXA, along with corresponding LV1 loadings plot (bottom).

**Figure 4:**
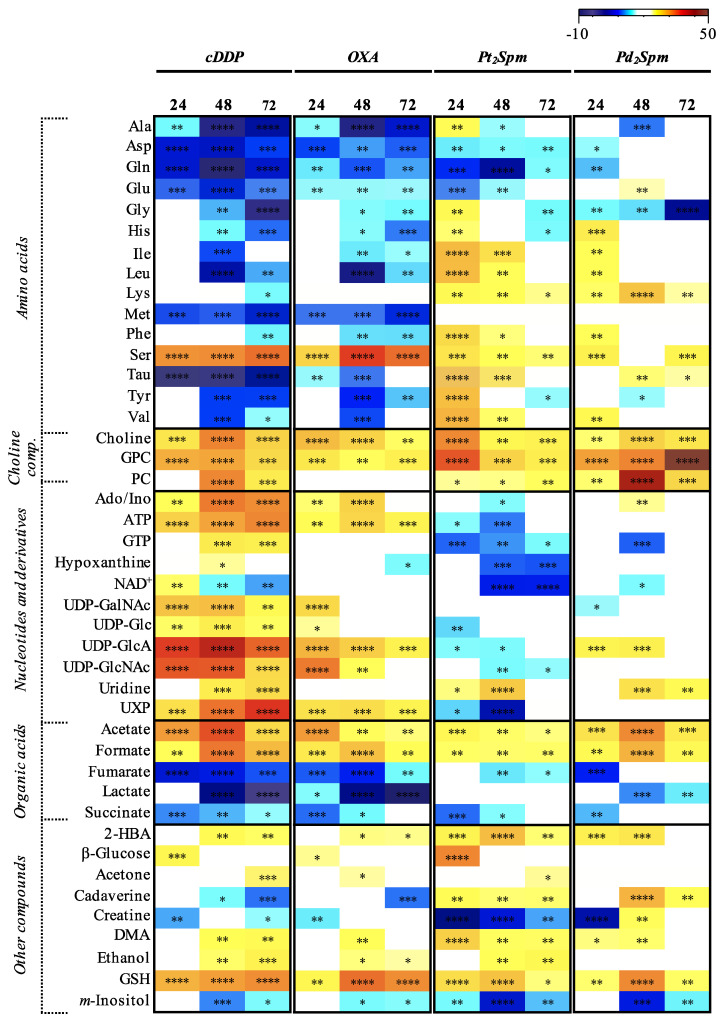
Heatmap of metabolites variations in aqueous extracts of MG-63 cells exposed to metal, in relation to controls (each time point was compared against the same time point of controls). The ES color-coded scale indicates increases and decreases of metabolite levels, in hot and cold colors, respectively. Inclusion criteria: |ES| > 0.50 and error < ES. ^✽^ *p*-value < 0.05; ^✽✽^ *p*-value < 0.01; ^✽✽✽^ *p*-value < 0.001; ^✽✽✽✽^ *p*-value < 0.0001. 3-Letter codes are used for amino acids. 2-HBA: 2-hydroxybutyrate; Ado: adenosine; ADP: adenosine diphosphate; ATP: adenosine triphosphate; DMA: dimethylamine; GPC: glycerophosphocholine; GSH: glutathione (reduced.); GTP: guanosine triphosphate; Ino: inosine; NAD^+^: nicotinamide adenine dinucleotide; PC: phosphocholine; UDP-GalNAc: uridine 5′-diphospho-*N*-acetylgalactosamine; UDP-Glc: uridine 5′-diphosphosphate glucose; UDP-GlcA: uridine 5′-diphosphoglucuronic acid; UDP-GlcNAc: uridine 5′-diphospho-*N*-acetylglucosamine; UXP: either of UDP/UTP/UDP-Glc/GalNAc.

Regarding the effects of exposure to cDDP, a relatively marked change in metabolic profile had been reported for lysed MG-63 cells [21,36,37] but an even stronger metabolic response is here observed, considering the cells’ polar extracts and expressed by the principal component analysis (PCA) of the corresponding time course ^1^H NMR spectra (Figure 3A, left). Supervised analysis through partial least squares discriminant analysis (PLS-DA) produced a robust model (Q^2^ = 0.933) (Figure 3A, right) with corresponding LV1 loadings expressing marked changes in lactate, amino acids, fumarate and formate, choline compounds and nucleotides (Figure 3A, bottom). These overall changes do not take into account the stepwise time variations, but the PLS-DA scores plot for cDDP exposure (Figure 3A, right) clearly shows that increasing exposure times tend to shift the samples towards more negative and positive LV1 and LV2, respectively. Control samples are not as sensitive to time within the 0–72 h range. Oxaliplatin also induces a strong effect on intracellular metabolome of MG-63 cells, as viewed both by PCA and PLS-DA (Q^2^ = 0.884), again with some suggestion of time course dependence in OXA-exposed cells, compared to controls (Figure 3B). Interestingly, the general profile of the corresponding PLS-DA plot seems similar to that obtained for cDDP, which suggests a similar metabolic response for both drugs. Peak integration and effect size calculation provide a time course evaluation of metabolite changes in MG-63 cells extracts due to cDDP and OXA (Tables S2 and S3). Results show that the metabolic signatures found were statistically robust for both drugs, since most changes survived the Bonferroni correction (^a^ in Tables S2 and S3). These metabolite changes are more easily read in a heatmap form (Figure 4, two left columns), where significant similarity is indeed noted in several respects: (i) general depletion of amino acids (as previously noted slightly for cDDP-treated lysed cells [21,37]), although more marked for cDDP, compared to OXA, with the exception of serine, which is always maintained significantly higher compared to controls, and with alanine, aspartate, glutamine, glutamate, methionine and taurine being taken up first (24 h) in both cases; (ii) increase in choline and GPC levels, although again more significantly for cDDP, while PC is increased at 48 and 72 h for cDDP only; (iii) consistent increases in acetate and formate, whereas fumarate, lactate and succinate are strongly decreased (particularly, the former two); (iv) variations in the levels of other compounds, namely, an initial increase in glucose which subsequently quickly levels off with control levels; consistently higher levels of GSH; decreased levels of *m*-inositol and creatine (also noted with statistical relevance in lysed cells [21,36,37]); and decrease in the biogenic amine cadaverine. Notably, nucleotides exhibit an apparent drug-specific signature distinguishing the effects of cDDP and OXA (Figure 4). Indeed, changes in guanosine triphosphate (GTP), hypoxanthine and nicotinamide adenine dinucleotide (NAD^+^) seem almost completely specific of cDDP, along with a more marked increased in several glycosylated uridine diphosphate (UDP) compounds, UXP species and uridine. In lysed MG-63 cells, an increase in UDP-GlcNAc had indeed been reported as part of the signature triggered by cDDP [21,37], although without statistical significance.

**Figure 5:**
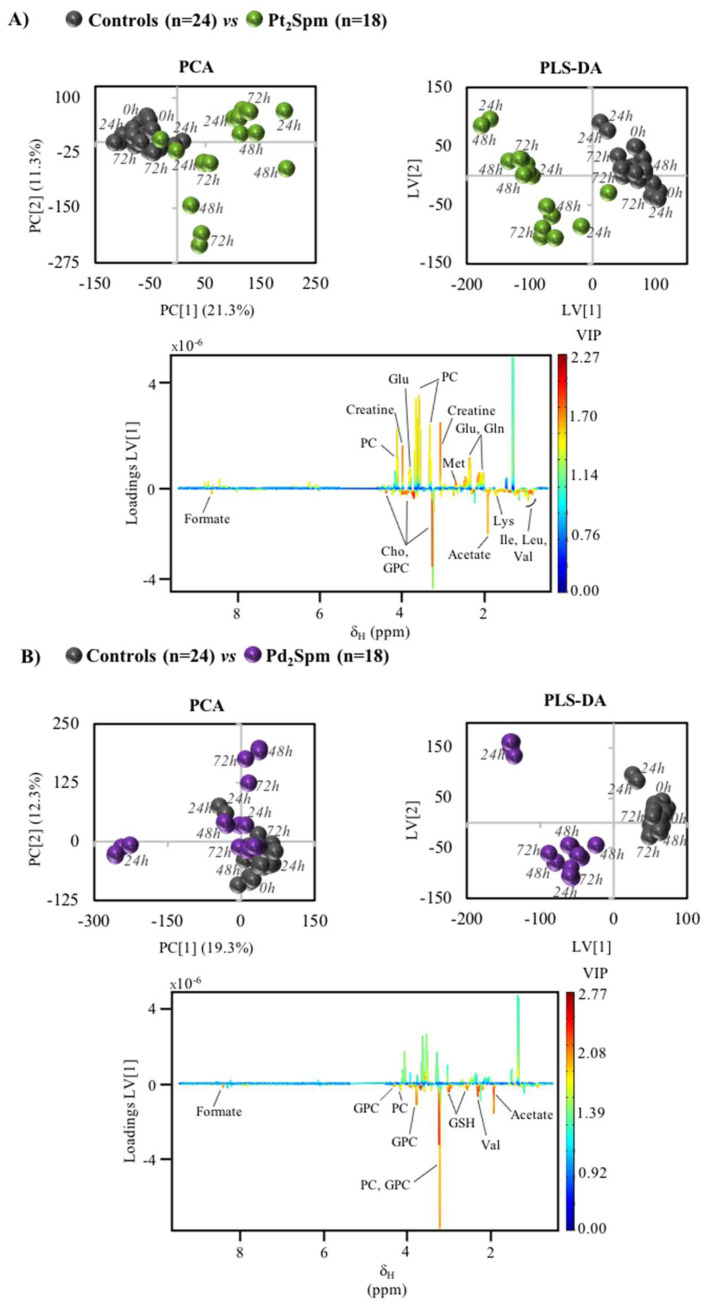
Multivariate analysis of MG-63 cells exposed to complexes Pt_2_Spm and Pd_2_Spm. (**A**) PCA (left) and PLS-DA (right, with LV = 2, R^2^_x_ = 0.290, R^2^_y_ = 0.935, Q^2^_cum_ = 0.857) scores plots obtained for cells exposed to 240 μM Pt_2_Spm, along LV1 loadings plot (bottom); (**B**) PCA (left) and PLS-DA (right, LV = 2, R^2^_x_ = 0.295, R^2^_y_ = 0.968, Q^2^_cum_ = 0.924) scores plots obtained for 24 µM Pd_2_Spm, along with corresponding LV1 loadings plot (bottom).

**Figure 6:**
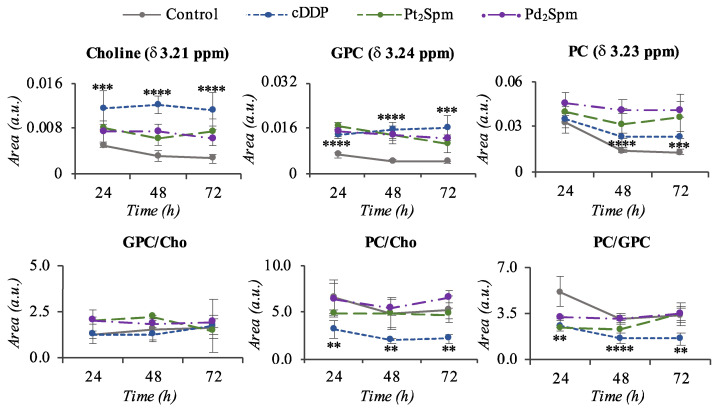
Relative changes in choline metabolite levels. Time course graphs (peak areas normalized by total spectral area, as a function of time) of choline metabolites and selected ratios, obtained for aqueous extracts of MG-63 cells under control conditions (grey solid line), exposed to 30 μM cDDP (blue dotted line), 240 μM Pt_2_Spm (green long dashed line) or 24 μM Pd_2_Spm (purple two-dashed line). ^✽✽^ *p*-value < 0.01; ^✽✽✽^ *p*-value < 0.001; ^✽✽✽✽^ *p*-value < 0.0001, however these indications are only indicated for cDDP, for the sake of clarity, and the authors are guided to Tables S2 to S5 or Figure 4 for complete information on statistical relevance. GPC: glycerophosphocholine; PC: phosphocholine.

In relation to the two Spm complexes under study, Pt_2_Spm and Pd_2_Spm, group separation from controls is also clearly observed (Figure 5A,B), although PCA results show overlap of some samples with controls. Notably, a time course tendency is not as clear as for the conventional drugs, for neither Spm complexes, while the profiles noted in the loading plots are distinctly different both from those found for cDDP and OXA, and between the Pt_2_Spm and Pd_2_Spm complexes themselves (Figure 5). In the corresponding metabolite variation tables (Tables S4 and S5) and their heatmap representation (Figure 4, right hand columns), it is visible that both Spm complexes trigger a clearly distinct response from amino acid metabolism compared to cDDP/OXA: indeed, most amino acids are now seen to increase (with the exception of alanine, aspartate, glutamine, glutamate and, in part, glycine, which match part of the early signature describing cDDP and OXA effects). Choline compounds and organic acids seem to behave similarly to cells exposed to the conventional drugs, in the sense that choline, GPC and PC levels are raised by all chelates compared to controls (Figure 6, top graphs). An exception relates to PC levels in the presence of the Spm complexes, which are more elevated by Pd_2_Spm, although not leading to significant differences in choline compound ratios (Figure 6). In addition, markedly distinct features are seen in regard to nucleotides, in the presence of the Spm complexes, namely comprising inverse (decreases) changes in adenosine triphosphate (ATP) and GTP, and distinct evolutions of the glycosylated UDP derivatives, while maintaining similar changes in GSH and *m*-inositol. Overall, the most significant distinguishing features between conventional Pt(II) drugs and the new Spm complexes relate to amino acid and nucleotides metabolisms, whereas the effects of Pt_2_Spm and Pd_2_Spm may be distinguished mainly with basis on the levels of nucleotides, lactate (not changed by Pt_2_Spm but slightly decreased by Pd_2_Spm), glucose (increased at 24 h only by Pt_2_Spm), creatine (consistently depleted by Pt_2_Spm, whereas Pd_2_Spm induces the reversal of an initial depletion at 24 h) and cadaverine (more markedly increased by Pd_2_Spm). A selection of compounds, the variations of which are plotted for controls, cDDP, Pt_2_Spm and Pd_2_Spm (Figure S1), shows how coincidently the two Spm complexes behave in many cases, with illustrated exceptions exemplified for cadaverine, GSH and tyrosine (the latter representing the general behavior of amino acids).

## 3. Discussion

These results demonstrate how cell extracts NMR metabolomics can provide exquisitely richer information on intracellular polar metabolism, compared to lysed cells. With such depth of information, correlations between drug cytotoxicity [23] and MG-63 cellular metabolic response to the complexes under study may be attempted. The IC_50_ trend recently reported for the same complexes followed the trend: cDDP ≈ Pd_2_Spm < OXA < Pt_2_Spm, confirming cDDP as highly cytotoxic against OS and revealing similar behavior for Pd_2_Spm, while indicating poor cytotoxicity for its Pt(II) analog [23]. The metabolomic results reported here indicate a clearly stronger impact of cDDP on cell metabolic profile, compared to the remaining three chelates, which suggests that the magnitude of metabolism response does not seem to relate to cytotoxicity in a straightforward manner, the qualitative nature of the resulting profile probably holding more information on such potential relationship.

### 3.1. MG-63 Cell Metabolism Associated to cDDP and OXA Treatment

Amino acids metabolism is a determinant feature in treated MG-63 cells and, for the conventional drugs, the general amino acid depletion, including of the anaplerotic amino acids glutamate (obtained through glutaminolysis, and converted to α-ketoglutarate by glutamate-dehydrogenase), aspartate (converted to oxaloacetate by aspartate transaminase), as well as methionine and taurine, is suggestive of activation of the tricarboxylic acid (TCA) cycle, probably to produce the higher levels of ATP noted and explaining the depletion in TCA cycle intermediates fumarate and succinate. Serine is an interesting exception to the general amino acid depletion behavior, possible explanations involving the need of replenishing glycine (through glycine hydroxymethyltransferase) and/or a need for enhanced synthesis of phosphatidylserine as recognized outer cell membrane indicator of apoptosis [42]. Confirmation of the latter hypothesis would recognize serine intracellular increase (observed for all chelates, although to a lesser extent for the spermine complexes) as a marker of this type of cell death. The activation of aerobic cellular energetic metabolism mentioned above is consistent with the strong depletion in lactate particularly from 48 h onwards, suggestive of a drug-induced shift from the enhanced glycolytic activity and lactate production characteristic of cancer cells (Warburg effect), to enhanced tricarboxylic acid cycle (TCA) and oxidative phosphorylation activity. This behavior is almost identical for cDDP and OXA, as are the changes in GSH (consistent high levels) and *m*-inositol (low levels), both compounds relatable to anti-oxidative stress mechanisms [43,44]. Notably, both conventional Pt(II) drugs also lead to depletion of cadaverine levels, an observation which is not straightforward to interpret, at this stage. In some cells (but to our knowledge not in cancer cells) biogenic amines, e.g., spermidine may react with GSH to form trypanothione [45]) and it is possible that other naturally occurring amines (including cadaverine) may undergo similar processes, thus somehow engaging with GSH metabolism. Both choline compounds and nucleotides signatures hold distinguishing features for the two conventional drugs. The more marked increases in choline GPC and PC for cDDP, along with the total absence of PC changes in OXA, suggest a distinct pattern of membrane membrane/biosynthesis (with, in the case of OXA, either no involvement of PC in membrane metabolism or a preferentially integration of PC into membrane lipids, both cases putatively explaining the absence of PC variation in OXA-treated cells). In addition, the nucleotides signature for OXA is much less eventful than for cDDP, with no involvement of GTP, NAD^+^ or uridine, and smaller increases in glycosylated UDP species, known to play important roles in post-translational glycosylation of proteins. This may be generally indicative of a mechanism with a less lasting impact on DNA. In particular, the early increase of glycosylated UDP derivatives in response to cDDP has been suggested [30] to be related to apoptotic cell death and, indeed, cDDP triggered an increase in UDP-GlcNAc in lysed MG-63 cells ([21,37] in which apoptosis was undergoing. It is possible that cell apoptosis (unfortunately not evaluated in this work) is less extensive in OXA-exposed cells, thus leading to less accumulation of these UDP derivatives. The relationship of these metabolites and serine, as potential markers of apoptosis, will require further investigation. In spite of the similarities noted for the metabolic impacts of cDDP and OXA on MG-63 cells, the above discussed distinguishing observations suggest possible mechanistic differences for OXA, namely regarding PC involvement in membrane metabolism and a lesser engagement of DNA. The latter point, in particular, would be consistent with the recent suggestion that OXA action may, in fact, be DNA-independent [46].

### 3.2. MG-63 Cell Metabolism Associated to Pt_2_Spm and Pd_2_Spm Treatment

One of the main metabolic differences between both Spm chelates and the conventional Pt(II) drugs is that the former induce a predominant accumulation of amino acids (except for the early depletion of anaplerotic alanine, aspartate, glutamate and glutamine), suggesting no or a smaller enhancement of TCA activity, giving rise to relatively small depletions in fumarate and succinate. Thus, amino acids do not seem to be feeding the TCA cycle as efficiently as for the conventional drugs, this being a feature of either Spm chelate, independently of the metal center. Indeed, this would explain the ATP depletion and the absence of ATP changes, for Pt_2_Spm and Pd_2_Spm, respectively. In addition, both Spm chelates seem to induce similar anti-oxidative mechanisms, judging by GSH and *m*-inositol levels. However, a switch is noted in cadaverine levels for both Spm chelates, with high levels of this amine suggesting that the presence of Spm may now play as a substitute of cadaverine in GSH metabolism. Indeed, a relationship has been reported between high (toxic) intracellular levels of Spm and GSH depletion [47], which draws attention to the complexity of biogenic amine metabolism and its intermingling with GSH metabolism.

It is interesting to note that Pd_2_Spm, which was previously found to be as cytotoxic as cDDP towards human OS [23], is the chelate that induces less changes in the cell endometabolome (consistently with previous observations on lysed MG-63 cells [21]), namely regarding amino acids and nucleotides, while all main choline membrane precursors are kept high, including PC (increased only slightly in Pt_2_Spm and not changing in OXA). This consistent elevation of membrane precursors in Pd_2_Spm-treated cells may indicate particularly extensive membrane degradation. Overall similarities between cDDP and Pd_2_Spm signatures (both chelates displaying high and similar cytotoxicity [23]), simultaneously distinguishing Pd_2_Spm and Pt_2_Spm (with opposing cytotoxicity levels), may hold a possible relationship with cytotoxicity. Such changes comprise a marked decrease in glycine at 72 h, high levels of all choline membrane precursors at all time points, increase in uridine and UDP-GlcNAc and depletion of lactate levels. We propose that this metabolic signature may be investigated further as holding a possible relationship to the similar IC_50_ values of cDDP and Pd_2_Spm, whereas the strong metabolic differences noted between the effects of the same complexes suggest that the exhibited similar cytotoxicity properties may be achieved through different metabolic mechanisms.

## 4. Materials and Methods

### 4.1. Chemicals and Solutions

Cisplatin (cis-dichlorodiammine platinum(II), cDDP, >99.9%), minimum essential medium (MEM), non-essential amino acids (NEAA), oxaliplatin (OXA), penicillin/streptomycin 100× solution, phosphate buffered saline (PBS), potassium tetrachloroplatinate(II) (K_2_PtCl_4_, >99.9%), potassium tetrachloropalladate(II) (K_2_PdCl_4_, >99.9%), *N*,*N*′**-bis(3-aminopropyl)-1,4-diaminobutane (spermine, free base, Spm), sodium pyruvate (100 mM), trypan blue (0.4% *w*/*v*), trypsin-EDTA (1×), as well as solvents, inorganic salts and acids (all of analytical grade) were purchased from Sigma-Aldrich Chemical S.A. (Sintra, Portugal). Fetal bovine serum (FBS) was obtained from Gibco-Life Technologies (Porto, Portugal). Pt_2_Spm and Pd_2_Spm were synthesized according to published procedures [48] optimized by the authors [16]. Briefly, 2 mmol of K_2_PdCl_4_ or K_2_PtCl_4_ were dissolved in a small amount of water, and 1 mmol Spm aqueous solution was added dropwise under stirring. After 24 h, the resulting powder was filtered and washed with acetone. For drug administration, initial stock solutions of cDDP (960 µM), OXA (1 mM), Pt_2_Spm (2.4 mM) and Pd_2_Spm (960 µM) were prepared in PBS, for the first two, and PBS/DMSO (20%), for the last two. All solutions were filtered (0.22 µm filter) and stored at 4 °C.

### 4.2. Cell Culture

The human osteosarcoma MG-63 cell line was obtained from ECACC (European Collection of Authenticated Cell Cultures, Salisbury, UK). Cells were grown as monolayers in MEM culture medium, supplemented with 10% (*v*/*v*) heat-inactivated FBS, 1% (*v*/*v*) sodium pyruvate, 1% (*v*/*v*) NEAA and antibiotics (penicillin/streptomycin 10×), and maintained under a humidified atmosphere of 5% CO_2_ at 37 °C.

### 4.3. Drug Administration and Sample Collection

MG-63 cell cultures were established in 24-well plates (1 mL/well) at a density of 4.0 × 10^4^ cells/cm^2^ to provide sufficiently high cell density for NMR analysis. After waiting 24 h for cells to adhere, the experiment was initiated (t = 0 h) by adding stock solutions of each drug to achieve the respective IC_50_ previously determined [23]: 30 µM cDDP, 100 μM OXA, 240 μM Pt_2_Spm or 24 μM Pd_2_Spm. According to the population doubling time for MG-63 (24 h), the 24, 48 and 72 h time-points after drug addiction were chosen for collection. At these time points, cells were harvested by trypsinization, washed with PBS (2×), centrifuged (at 1100 rpm, for 5 min) and counted by the trypan blue assay. The obtained cell pellets were then snap frozen in liquid nitrogen and stored at −80 °C until cell extraction for NMR analysis. Three independent experiments with duplicates for each condition (time point and drug) were performed.

### 4.4. Cell Extraction for Sample Preparation

Intracellular aqueous metabolites were extracted using a dual-phase extraction, with methanol/chloroform/water, as described elsewhere [49,50]. Briefly, 650 µL of cold methanol 80% was added to cell pellets, quickly vortexed, transferred into microcentrifuge tubes containing 0.5 mm glass beads, to aid cell breakage, followed by 5 min vortexing. Cold chloroform (260 μL + 260 μL) and cold milliQ water (220 μL) were then added to each sample, each addition being followed by 5 min vortexing, and the samples were left to rest on ice for 10 min. After centrifugation (at 2000× *g*, for 15 min), the upper aqueous phase was then carefully transferred into new vials, dried under vacuum and stored at −80 °C until analysis.

### 4.5. NMR Spectroscopy

NMR spectra acquisition was conducted on dried aqueous extracts after reconstitution in 600 μL of deuterated phosphate buffer (100 mM phosphate, pH 7.4) previously prepared in (99.9% deuterium) with 60 mM Na_2_HPO_4_, 40 mM NaH_2_PO_4_ and 0.1 mM 3-(trimethylsilyl)-propionic-2,2,3,3-d4 acid (TSP) for chemical shift referencing. After vortex homogenization, a 550 μL volume of each sample was transferred into 5 mm NMR tubes. NMR spectra were acquired on a Bruker Avance DRX-500 spectrometer (Bruker, Ettlingen, Germany) operating at 500.13 MHz for ^1^H observation, at 298 K, using a 5 mm probe. Standard 1D ^1^H NMR spectra with water presaturation (*noesypr1d* pulse program in the Bruker library) were recorded with a 7002.801 Hz spectral width, 32 k data points, a 4 s relaxation delay (d1) and 512 scans. Each free-induction decay was zero-filled to 64 k points and multiplied by a 0.3 Hz exponential function before Fourier transformation. Unidimensional ^1^H NMR spectra were manually phased, baseline-corrected and chemical shift referenced to TSP signal at δ 0 ppm. 2D ^1^H/^1^H total correlation (TOCSY), ^1^H/^13^C heteronuclear single quantum correlation (HSQC), and *J*-resolved (*J*-res) spectra were also acquired for selected samples to assist spectral assignment, which was also based on comparison with existing literature [51,52] and data available on spectral databases, such as the Bruker BIOREFCODE database and the human metabolome database (HMDB) [53].

### 4.6. Data Processing and Statistics

1D ^1^H NMR spectra were converted into matrices (AMIX-viewer 3.9.14, Bruker Biospin, Rheinstetten, Germany), after exclusion of the spectral regions related to water suppression (δ 4.55–5.40) and methanol contamination (singlet at δ 3.36). Spectra were aligned using a recursive segment-wise peak alignment (Matlab 8.3.0, The MathWorks Inc., Natick, MA, USA), to minimize chemical shift variations [54] and normalized to total spectral area, which accounts for differences regarding sample concentration. After unit variance (UV) scaling (SIMCA-P 11.5; Umetrics, Umeå, Sweden) [55], multivariate analysis was carried out using principal component analysis (PCA), an unsupervised method used to detect intrinsic clusters and outliers within the data set, and partial-least-squares discriminant analysis (PLS-DA), a supervised method to maximize class discrimination. PLS-DA models were considered statistically robust for predictive power (Q^2^) values ≥ 0.50. The relevant resonances identified in PLS-DA loadings plots, together with selected signals in the 1D spectrum, were integrated (Amix 3.9.5, Bruker BioSpin, Rheinstetten, Germany), normalized to total spectral area, and variations assessed by univariate analysis (Shapiro–Wilk test to assess data normality, Student’s *t*-test or Wilcoxon test for normally-distributed or non-normally distributed data, respectively) (Python 3.7.8, Python Software Foundation, Fredericksburg, VA, USA). The individual metabolites that most contributed to class separation were selected based on their statistical significance (*p* < 0.05, effect size [56], |ES| > 0.5 and ES error < 75%) and expressed in a heatmap colored as a function of ES (R 4.0.2, R Foundation for Statistical Computing, Vienna, Austria). Bonferroni correction [57] was used to correct *p*-values for multiple comparisons.

## 5. Conclusions

The present results revealed detailed metabolic signatures descriptive of the impact of conventional and potential new Pt(II) and Pd(II) anticancer agents on the endometabolome of MG-63 human osteosarcoma cells. Interestingly, the two most cytotoxic chelates, cDDP and Pd_2_Spm, exhibited significantly distinct signatures (both qualitatively and quantitatively), namely regarding amino acids and nucleotides metabolisms, with membrane degradation processes seemingly more enhanced for Pd_2_Spm, in tandem with lower TCA and oxidative phosphorylation activation and lesser reversal of the Warburg effect. Raised serine levels and glycosylated UDP species may be suggestive of ongoing apoptotic behavior and, if so, interestingly enough, apoptotic cell death appears to be less marked for both Pt(II) and Pd(II) Spm complexes, as compared to conventional mononuclear Pt(II) agents such as cDDP. This suggests that high cytotoxicity may be achievable through significantly distinct mechanisms, strongly depending on metal/ligand type and number of metal centers, an aspect which requires further investigation. This knowledge may unveil metabolic signatures as candidate markers of drug performance (degree of cytotoxicity and resistance), with potential translation to in vivo and clinical applications.

## Supplementary Materials

Table S1: List of metabolites and corresponding spin systems identified in the 500 MHz ^1^H NMR spectra of aqueous extracts of OS MG-63 cells; Table S2: Metabolite variations in MG-63 cells exposed to 30 µM cDDP at 24, 48 and 72 h, compared to controls; Table S3: Metabolite variations in MG-63 cells exposed to 100 µM OXA at 24, 48 and 72 h, compared to controls; Table S4: Metabolite variations in MG-63 cells exposed to 240 µM Pt_2_Spm at 24, 48 and 72 h, compared to controls; Table S5: Metabolite variations in MG-63 cells exposed to 24 µM Pd_2_Spm at 24, 48 and 72 h, compared to controls; Figure S1: Time course changes for selected metabolites in MG-63 cells exposed to Pt_2_Spm and Pd_2_Spm, compared to cDDP and controls. Graphs represent peak areas normalized by total spectral area, as a function of time.

## Sample Availability

Samples of the compounds containing spermine are not directly available form the suthors but may be synthesized following the instructions referred to in Section 4.1.
