# Supplementary material for: Response of Osteosarcoma Cell Metabolism to Platinum and Palladium Chelates as Potential New Drugs
Source: Molecules. 2021 Aug 8;26(16):4805. doi: 10.3390/molecules26164805 (PMC8401043; doi:10.3390/molecules26164805)
Supplement: Supplementary file 1 [file molecules-26-04805-s001.zip › molecules-1317181-supplementary-RC .pdf]

molecules

Article SUPPLEMENTARY INFORMATION

Response of Osteosarcoma Cell Metabolism to Platinum and Palladium Chelates as Potential New Drugs

Ana S. Martins [1,2], Ana L. M. Batista de Carvalho [2], Maria P. M. Marques [2,3,*] and Ana M. Gil [1,*]

1 CICECO – Aveiro Institute of Materials (CICECO/UA), Department of Chemistry, University of Aveiro, Campus Universitário de Santiago, 3810-193 Aveiro, Portugal
2 Unidade de I&D Química-Física Molecular, Department of Chemistry, University of Coimbra, Rua Larga, 300-535 Coimbra, Portugal
3 Department of Life Sciences, Faculty of Science and Technology, University of Coimbra, Calçada Martim de Freitas, 3000-456 Coimbra, Portugal
* Correspondence: agil@ua.pt (A.M.G.); pmc@ci.uc.pt (M.P.M.M.)

Table S1. List of metabolites and corresponding spin systems identified in the 500 MHz ^{1}H NMR spectra of aqueous extracts of OS MG-63 cells. Multiplicity: *s*, singlet; *d*, doublet; *t*, triplet; *q*, quartet; *m*, multiplet; *dd*, double doublet; *br*, broad. 2-HBA: 2-hydroxybutyrate; Ado: adenosine; ADP: adenosine diphosphate; ATP: adenosine triphosphate; DMA: dimethylamine; GPC: glycerophosphocholine; GSH: glutathione (reduced); GTP: guanosine triphosphate; Ino: inosine; NAD$^+$: nicotinamide adenine dinucleotide; PC: phosphocholine; UDP-GalNAc: uridine 5′-diphospho-*N*-acetylgalactosamine; UDP-Glc: uridine 5′-diphosphosphate glucose; UDP-GlcA: uridine 5′-diphosphoglucuronic acid; UDP-GlcNAc: uridine 5′-diphospho-*N*-acetylglucosamine. Spectral assignment was based on comparison with existing literature [51-52] and data available on spectral databases, such as the Bruker BIOREFCODE database and the human metabolome database (HMDB) [53].

Metabolite assignment	δ_H ppm (multiplicity, assignment)
Amino acids	
Alanine	1.48 (d, β-CH$_3$); 3.78 (q, α-CH)
Aspartate	2.68 (dd, β-CH); 2.82 (dd, β′-CH); 3.90 (dd, α-CH)
Glutamate	2.05 (m, β-CH$_2$); 2.13 (m, β′-CH$_2$); 2.36 (m, γ-CH$_2$); 3.75 (dd, α-CH)
Glutamine	2.14 (m, β-CH$_2$); 2.47 (m, γ-CH$_2$); 3.78 (t, α-CH)
Glycine	3.56 (s, α-CH$_2$)
Histidine	7.08 (s, C4H, ring)
Isoleucine	0.94 (t, δ-CH$_3$); 1.02 (d, γ-CH3)
Leucine	0.96 (t, δ-CH$_3$); 1.71 (m, β-CH$_2$/γ-CH)
Lysine	1.47 (m, γ-CH$_2$); 1.73 (m, δ-CH$_2$); 1.92 (m, β-CH$_2$); 3.02 (m, ε-CH$_2$); 3.76 (t, α-CH);
Methionine	2.65 (t, γ-CH$_2$)
Phenylalanine	3.14 (m, β-CH); 3.99 (m, α-CH); 7.33 (m C2H)/C6H, ring); 7.39 (m, C4H, ring); 7.43 (m, C3H/C5H, ring)
Serine	3.85 (dd, α-CH); 3.97 (m, β-CH$_2$); 3.99 (dd, β′-CH$_2$)
Taurine	3.26 (t, S-CH$_2$); 3.42 (t, N-CH$_2$)
Tyrosine	3.07 (m, β′-CH$_2$); 3.21 (m, β-CH$_2$); 3.96 (m, α-CH); 6.91 (d, C3H/C5H, ring); 7.20 (d, C2H/C6H, ring)
Valine	0.99 (d, γ-CH$_3$); 1.05 (d, γ′-CH$_3$); 2.28 (m, β-CH)
Choline compounds	
Choline	3.21 (s, N(CH$_3$)$_3$); 3.53 (m, β-CH$_2$(NH)); 4.07 (m, α-CH$_2$(OH))
GPC	3.24 (s, N(CH$_3$)$_3$); 3.94 (m, α-CH$_2$); 4.33 (m, α-CH$_2$(PO$_3$))
PC	3.22 (s, N(CH$_3$)$_3$); 3.60 (m, N-CH$_2$); 4.17 (m, PO$_3$-CH$_2$)
Nucleotides and derivatives	
Ado/Ino	4.28 (q, C4′H, ribose); 4.44 (dd, C3′H, ribose); 6.10 (d, C1′H, ribose); 8.24 (s, C2H, ring); 8.35 (s, C8H, ring)
ADP	4.22 (m, C5′H, ribose); 4.38 (m, C4′H); 6.15 (d, C1′H, ribose); 8.28 (s, C2H, ring); 8.52 (s, C8H, ring)
ATP	4.22 (m, C5′H, ribose); 4.39 (m, C4′H); 6.14 (d, C1′H, ribose); 8.27 (s, C2H, ring); 8.54 (s, C8H, ring)
GTP	5.94 (d, C1′H, ribose), 8.14 (s, C8H, ring)
NAD$^+$	8.18 (s, A2); 8.43 (s, A8), 8.83 (d, N4); 9.15 (d, N6); 9.34 (s, N2)
UDP-GalNAc	5.55 (m, C1H, Gal)
UDP-Glc	5.61 (dd); 5.99 (d); 7.95 (d, C6H, ring)
UDP-GlcA	5.97 (d); 7.96 (d, C6H, ring)
UDP-GlcNAc	5.52 (m, C1H, Glc)

Uridine	5.90 (d, C5H, ring); 7.87 (d, C6H, ring)
Organic acids	
Acetate	1.92 (s, β-CH_3)
Acetone	2.23 (s, CH_3)
Formate	8.46 (s, CH)
Fumarate	6.52 (s, HC=CH)
Lactate	1.33 (d, CH_3), 4.10 (q, CH)
Succinate	2.40 (s, α-CH_2/β-CH_2)
Other compounds	
2-HBA	0.89 (t, γ-CH_3); 1.64 (m, β-CH_2); 1.73 (m, β′-CH_2)
β-Glucose	3.23 (dd, C2H); 3.41 (t, C4H); 3.47 (m, C5H); 3.49 (t, C3H); 3.71 (m, C3H); 5.24 (d, C1H)
Cadaverine	1.46 (m, γ-CH2); 1.71 (m, β-CH2); 3.02 (t, α-CH_2)
Creatine	3.04 (s, N-CH_3); 3.93 (s, N-CH_2)
DMA	2.73 (s, CH_3)
Ethanol	1.18 (m, β-CH_3)
GSH	2.17 (m, β-CH_2 Glu); 2.56 (m, γ-CH_2 Glu); 2.96 (m, β-CH_2 Cys); 3.79 (m, α-CH Glu)
Hypoxanthine	8.20 (s, C2H, ring); 8.21 (s, C8H)
m-Inositol	3.28 (t, C5H); 3.53 (dd, C1H/C3H); 3.63 (t, C4H/C6H); 4.06 (t, C2H)

Table S2. Metabolite variations in MG-63 cells exposed to 30 μM cDDP at 24, 48, and 72 h, compared to controls. Only statistically significant differences are shown (*p*-value < 0.05), also compliant with effect size modulus ∣ES∣ > 0.50, ES error < 75%. 3-Letter codes are used for amino acids; UXP: either of UDP/UTP/UDP-Gal/GlcNAc species; other abbreviations as defined in Table S1.

**Metabolite**	**δ_H ppm (multiplicity)**	**cDDP**					
		**24 h**		**48 h**		**72h**	
		**ES ± Error**	***p*-value**	**ES ± Error**	***p*-value**	**ES ± Error**	***p*-value**
Amino acids							
Ala	1.48 (d)	-1.95 ± 1.38	7.0 x 10^{-3}	-6.62 ± 2.88 [a,b]	4.5 x 10^{-7}	-5.00 ± 2.30 [a,b]	4.8 x 10^{-6}
Asp	2.68 (dd)	-4.11 ± 2.00 [a]	3.9 x 10^{-5}	-4.37 ± 2.08 [a]	1.0 x 10^{-5}	-3.19 ± 1.71 [a]	2.5 x 10^{-4}
Gln	2.47 (m)	-4.16 ± 2.01 [a]	2.9 x 10^{-5}	-9.00 ± 3.77 [a]	1.3 x 10^{-5}	-4.23 ± 2.04 [a]	2.5 x 10^{-5}
Glu	2.36 (m)	-2.91 ± 1.62 [a,b]	5.0 x 10^{-4}	-3.89 ± 1.92 [a,b]	5.8 x 10^{-5}	-2.74 ± 1.58 [a,b]	7.8 x 10^{-4}
Gly	3.56 (s)	–	–	-2.42 ± 1.49 [a,b]	1.9 x 10^{-3}	-6.92 ± 1.53 [a,b]	2.9 x 10^{-7}
His	7.08 (s)	–	–	-1.84 ± 1.35 [c]	9.6 x 10^{-3}	-2.87 ± 1.61 [a]	5.6 x 10^{-4}
Ile	0.94 (t)	–	–	-3.10 ± 1.68 [a,c]	3.2 x 10^{-4}	–	–
Leu	0.96 (t)	–	–	-4.84 ± 2.24 [a,b]	7.7 x 10^{-6}	-2.48 ± 1.50 [a]	1.6 x 10^{-3}
Lys	1.92 (m)	–	–	–	–	–	–
Met	2.65 (t)	-3.12 ± 1.69 [a]	3.2 x 10^{-4}	-2.99 ± 1.65 [a]	4.0 x 10^{-4}	-4.08 ± 1.99 [a]	4.1 x 10^{-5}
Phe	7.33 (m)	–	–	–	–	-2.00 ± 1.39	6.0 x 10^{-3}
Ser	3.97 (dd)	6.38 ± 2.79 [a,b]	6.3 x 10^{-7}	6.65 ± 2.89 [a,c]	4.3 x 10^{-7}	8.32 ± 3.52 [a]	4.8 x 10^{-8}
Tau	3.42 (t)	-7.92 ± 3.36 [a,b]	8.2 x 10^{-8}	-7.25 ± 3.11 [a,b]	1.9 x 10^{-7}	-5.08 ± 2.33 [a,b]	5.0 x 10^{-6}
Tyr	6.91 (d)	–	–	-3.18 ± 1.70 [a,b]	2.6 x 10^{-4}	-3.18 ± 1.70 [a]	2.6 x 10^{-4}
Val	1.05 (d)	–	–	-3.19 ± 1.71 [a,c]	2.5 x 10^{-4}	–	–
Choline compounds							
Choline	3.21 (s)	3.27 ± 1.73 [a,b]	1.6 x 10^{-3}	7.16 ± 3.08 [a,b]	2.2 x 10^{-7}	4.02 ± 1.97 [a]	3.9 x 10^{-5}
GPC	3.24 (s)	5.36 ± 2.42 [a,b]	3.2 x 10^{-6}	5.71 ± 2.55 [a,b]	1.8 x 10^{-6}	3.82 ± 1.90 [a,b]	5.8 x 10^{-5}
PC	3.22 (s)	–	–	6.97 ± 3.01 [a,c]	2.7 x 10^{-7}	3.10 ± 1.68 [a,b]	3.3 x 10^{-4}
Nucleotides and derivatives							
Ado/Ino	4.28 (q)	2.37 ± 1.48 [a]	2.1 x 10^{-3}	7.66 ± 3.27 [a,c]	1.1 x 10^{-7}	6.54 ± 2.85 [a]	4.9 x 10^{-7}
ATP	4.39 (m)	4.07 ± 1.98 [a,c]	3.5 x 10^{-5}	5.77 ± 2.57 [a,c]	1.6 x 10^{-6}	7.02 ± 3.03 [a]	2.6 x 10^{-7}
GTP	5.94 (d)	–	–	3.11 ± 1.68 [a]	3.0 x 10^{-4}	2.97 ± 1.64 [a]	4.3 x 10^{-4}
NAD^+	8.43 (s)	1.98 ± 1.38	6.4 x 10^{-3}	-1.81 ± 1.34	9.8 x 10^{-3}	-2.53 ± 1.52 [a]	1.4 x 10^{-3}
UDP-GalNAc	5.55 (m)	4.52 ± 2.13 [a,b]	1.4 x 10^{-5}	4.79 ± 2.23 [a,b]	8.5 x 10^{-6}	2.62 ± 1.54 [a]	1.0 x 10^{-3}
UDP-Glc	5.61 (m)	2.00 ± 1.39	6.0 x 10^{-3}	2.99 ± 1.65 [a]	4.1 x 10^{-4}	2.11 ± 1.41 [a]	4.5 x 10^{-3}
UDP-GlcA	7.96 (d)	11.89 ± 4.89 [a]	3.4 x 10^{-9}	17.22 ± 6.98 [a]	4.2 x 10^{-11}	9.04 ± 3.79 [a]	1.3 x 10^{-5}
UDP-GlcNAc	5.52 (m)	9.95 ± 4.14 [a,b]	9.1 x 10^{-9}	9.85 ± 4.10 [a,b]	5.7 x 10^{-6}	3.75 ± 1.88 [a]	6.2 x 10^{-5}
Uridine	7.87 (d)	–	–	3.04 ± 1.66 [a,b]	3.7 x 10^{-4}	3.65 ± 1.84 [a]	7.1 x 10^{-5}
UXP	5.99 (m)	3.56 ± 1.82 [a]	7.9 x 10^{-5}	8.10 ± 3.43 [a,b]	2.5 x 10^{-5}	12.96 ± 5.31 [a]	6.9 x 10^{-10}
Organic acids							

Acetate	1.92 (s)	6.06 ± 2.68 [a]	1.0 x 10^{-7}	10.48 ± 4.34 [a,b]	4.6 x 10^{-7}	6.00 ± 2.66 [a]	1.1 x 10^{-7}
Formate	8.46 (s)	2.27 ± 1.45	7.9 x 10^{-3}	7.91 ± 3.36 [a]	2.0 x 10^{-5}	4.89 ± 2.26 [a]	7.5 x 10^{-6}
Fumarate	6.52 (s)	-4.10 ± 1.99 [a]	3.3 x 10^{-5}	-3.81 ± 1.90 [a]	6.4 x 10^{-5}	-3.04 ± 1.66 [a]	3.7 x 10^{-4}
Lactate	1.33 (d)	–	–	-5.51 ± 2.48 [a,b]	3.0 x 10^{-6}	-7.49 ± 3.20 [a,b]	4.5 x 10^{-8}
Succinate	2.40 (s)	-2.67 ± 1.56 [a]	9.4 x 10^{-4}	-2.40 ± 1.48 [a,b]	1.9 x 10^{-3}	–	–
Other compounds							
2-HBA	0.89 (t)	–	–	2.51 ± 1.51 [a]	1.5 x 10^{-3}	2.06 ± 1.40	5.2 x 10^{-3}
β-Glucose	5.24 (d)	2.67 ± 1.56 [a]	9.4 x 10^{-4}	–	–	–	–
Acetone	2.23 (s)	–	–	–	–	2.73 ± 1.57 [a]	7.8 x 10^{-4}
Cadaverine	3.02 (t)	–	–	–	–	-2.83 ± 1.60 [a]	6.2 x 10^{-4}
Creatine	3.04 (s)	-2.49 ± 1.51 [a,b]	1.5 x 10^{-3}	–	–	–	–
DMA	2.73 (s)	–	–	2.40 ± 1.48 [a]	1.9 x 10^{-3}	2.56 ± 1.53 [a]	1.0 x 10^{-3}
Ethanol	1.18 (t)	–	–	1.96 ± 1.38	6.9 x 10^{-3}	2.84 ± 1.60 [a]	6.1 x 10^{-4}
GSH	3.79 (m)	5.19 ± 2.37 [a]	4.2 x 10^{-6}	5.97 ± 2.64 [a,c]	7.3 x 10^{-5}	6.54 ± 2.85 [a]	5.0 x 10^{-7}
m-Inositol	4.06 (t)	–	–	-2.71 ± 1.57 [a,b]	8.4 x 10^{-4}	-1.64 ± 1.31	2.5 x 10^{-2}

[a] Metabolite variation remaining significant after Bonferroni correction [57]; [b] metabolite variations reported previously for cDDP-treated cancer cells [21,27,28,30,36,37]; [c] metabolite variations noted in different direction compared to previous reports for cDDP-treated cancer cells [21,27,28,30,36,37].

Table S3. Metabolite variations in MG-63 cells exposed to 100 µM OXA at 24, 48, and 72 h, compared to controls. Only statistically significant differences are shown (*p*-value < 0.05), also compliant with effect size modulus |ES| > 0.50, ES error < 75%. 3-Letter codes are used for amino acids; UXP: either of UDP/UTP/UDP-Gal/GlcNAc species; other abbreviations as defined in Table S1.

**Metabolite**	**δ_H ppm (multiplicity**	**OXA**					
		**24 h**		**48 h**		**72h**	
		**ES ± Error**	***p*-value**	**ES ± Error**	***p*-value**	**ES ± Error**	***p*-value**
Amino acids							
Ala	1.48 (d)	–	–	-6.52 ± 2.84 [a]	5.2 x 10^{-7}	-4.21 ± 2.03 [a]	2.6 x 10^{-5}
Asp	2.68 (dd)	-3.14 ± 1.69 [a]	2.9 x 10^{-4}	-2.55 ± 1.52 [a]	1.2 x 10^{-3}	-2.94 ± 1.63 [a]	4.5 x 10^{-4}
Gln	2.47 (m)	-2.08 ± 1.40 [d]	4.8 x 10^{-3}	-3.02 ± 1.66 [a,d]	3.9 x 10^{-4}	-2.53 ± 1.52 [a]	1.4 x 10^{-3}
Glu	2.36 (m)	-1.94 ± 1.37 [d]	7.1 x 10^{-3}	-1.97 ± 1.38 [d]	6.8 x 10^{-3}	–	–
Gly	3.56 (s)	–	–	–	–	-1.81 ± 1.34	9.8 x 10^{-3}
His	7.08 (s)	–	–	–	–	-2.77 ± 1.58 [a]	7.3 x 10^{-4}
Ile	0.94 (t)	–	–	-2.08 ± 1.41	4.8 x 10^{-3}	–	–
Leu	0.96 (t)	–	–	-6.19 ± 2.72 [a]	8.3 x 10^{-7}	-2.17 ± 1.43 [a]	3.8 x 10^{-3}
Met	2.65 (t)	-2.83 ± 1.60 [a]	6.4 x 10^{-4}	-2.82 ± 1.60 [a]	6.4 x 10^{-4}	-3.74 ± 1.87 [a]	6.9 x 10^{-5}
Phe	7.33 (m)	–	–	-2.14 ± 1.42 [a,b]	4.1 x 10^{-3}	-2.26 ± 1.45 [a]	2.9 x 10^{-3}
Ser	3.97 (dd)	4.01 ± 1.96 [a]	4.0 x 10^{-5}	12.08 ± 4.96 [a]	1.4 x 10^{-9}	8.47 ± 3.57 [a]	2.8 x 10^{-8}
Tau	3.42 (t)	-1.88 ± 1.36	8.7 x 10^{-3}	-2.79 ± 1.59 [a]	7.0 x 10^{-4}	–	–
Tyr	6.91 (d)	–	–	-3.46 ± 1.79 [a]	1.3 x 10^{-4}	-2.33 ± 1.47 [a]	2.1 x 10^{-3}
Val	1.05 (d)	–	–	-3.10 ± 1.68 [a]	3.1 x 10^{-3}	–	–
Choline compounds							
Choline	3.21 (s)	4.84 ± 2.24 [a,c]	7.7 x 10^{-6}	4.03 ± 1.97 [a]	3.8 x 10^{-5}	2.09 ± 1.41 [a]	4.7 x 10^{-3}
GPC	3.24 (s)	3.24 ± 1.72 [a]	1.9 x 10^{-4}	2.23 ± 1.44 [a]	2.4 x 10^{-3}	2.92 ± 1.62 [a]	4.6 x 10^{-4}
Nucleotides and derivatives							
Ado/Ino	4.28 (q)	1.86 ± 1.36	9.0 x 10^{-3}	3.96 ± 1.94 [a]	4.5 x 10^{-5}	–	–
ATP	4.39 (m)	2.45 ± 1.49 [a]	1.7 x 10^{-3}	3.84 ± 1.91 [a]	5.8 x 10^{-5}	2.82 ± 1.60 [a]	6.4 x 10^{-4}
UDP-GalNAc	5.55 (m)	3.77 ± 1.88 [a]	6.6 x 10^{-5}	–	–	–	–
UDP-GlcA	7.96 (d)	5.12 ± 2.34 [a]	3.9 x 10^{-6}	4.17 ± 2.02 [a]	3.2 x 10^{-5}	2.85 ± 1.61 [a]	6.2 x 10^{-4}
UDP-GlcNAc	5.52 (m)	7.66 ± 3.27 [a]	1.1 x 10^{-7}	2.26 ± 1.45 [a]	2.9 x 10^{-3}	–	–
UXP	5.99 (m)	3.39 ± 1.77 [a]	1.2 x 10^{-4}	3.63 ± 1.84 [a]	7.9 x 10^{-5}	2.82 ± 1.60 [a]	6.4 x 10^{-4}
Organic acids							
Acetate	1.92 (s)	6.12 ± 2.70 [a]	9.0 x 10^{-7}	2.27 ± 1.45 [a]	2.8 x 10^{-3}	–	–
Formate	6.52 (s)	3.16 ± 1.70 [a]	2.7 x 10^{-4}	4.66 ± 2.18 [a,c]	1.5 x 10^{-5}	2.42 ± 1.49 [a]	1.9 x 10^{-3}
Fumarate	1.33 (d)	-2.97 ± 1.64 [a,d]	4.3 x 10^{-4}	-3.59 ± 1.83 [a]	9.4 x 10^{-5}	-1.95 ± 1.37	7.0 x 10^{-3}
Lactate	2.40 (s)	–	–	-5.41 ± 2.44 [a]	2.9 x 10^{-6}	-9.64 ± 4.02 [a]	1.2 x 10^{-8}

Succinate	1.92 (s)	-2.87 ± 1.61 [a]	5.8 x 10^{-4}	-1.96 ± 1.38	7.0 x 10^{-3}	–	–
Other compounds							
Cadaverine	3.02 (t)	–	–	–	–	-2.82 ± 1.60 [a]	6.4 x 10^{-4}
Creatine	3.04 (s)	-2.07 ± 1.40	5.0 x 10^{-3}	–	–	–	–
DMA	2.73 (s)	–	–	2.32 ± 1.46 [a]	2.2 x 10^{-3}	–	–
GSH	3.79 (m)	2.64 ± 1.55 [a]	3.0 x 10^{-3}	8.37 ± 3.54 [a,c]	4.8 x 10^{-8}	6.63 ± 2.88 [a]	4.4 x 10^{-7}

[a] Metabolite variation remaining significant after Bonferroni correction [57]; [b] newly identified variations compared to cDDP-treated MG-63 cells; [c] metabolite variations reported previously for OXA-treated cancer cells [38,58]; [d] metabolite variations noted in different direction compared to previous reports for OXA-treated cancer cells [38,58].

Table S4. Metabolite variations in MG-63 cells exposed to 240 µM Pt_2Spm at 24, 48, and 72 h, compared to controls. Only statistically significant differences are shown (p-value < 0.05), also compliant with effect size modulus |ES| > 0.50, ES error < 75%. 3-Letter codes are used for amino acids; UXP: either of UDP/UTP/UDP-Gal/GlcNAc species; other abbreviations as defined in Table S1.

**Metabolite**	**δ_H ppm (multiplicity**	**Pt_2Spm**					
		**24 h**		**48 h**		**72h**	
		**ES ± Error**	***p*-value**	**ES ± Error**	***p*-value**	**ES ± Error**	***p*-value**
Amino acids							
Ala	1.48 (d)	2.48 ± 1.50 [a]	1.6 x 10^{-3}	–	–	–	–
Asp	2.68 (dd)	-2.07 ± 1.40	5.0 x 10^{-3}	–	–	-1.80 ± 1.34	9.9 x 10^{-3}
Gln	2.47 (m)	-3.34 ± 1.75 [a]	1.7 x 10^{-4}	-5.06 ± 2.32 [a]	4.4 x 10^{-6}	–	–
Glu	2.36 (m)	-2.72 ± 1.57 [a]	8.0 x 10^{-4}	-1.80 ± 1.34 [a]	9.9 x 10^{-3}	–	–
Gly	3.56 (s)	2.62 ± 1.54 [a,b,c]	1.1 x 10^{-3}	–	–	-1.88 ± 1.36	8.7 x 10^{-3}
His	7.08 (s)	1.86 ± 1.35 [b,c]	9.3 x 10^{-3}	–	–	–	–
Ile	0.94 (t)	4.91 ± 2.27 [a,b,c]	6.8 x 10^{-6}	3.47 ± 1.79 [a]	6.6 x 10^{-4}	–	–
Leu	0.96 (t)	4.92 ± 2.27 [a,b,c]	6.7 x 10^{-6}	2.30 ± 1.46	9.3 x 10^{-3}	–	–
Lys	1.92 (m)	2.59 ± 1.53 [a]	2.0 x 10^{-3}	2.31 ± 1.44	9.1 x 10^{-3}	–	–
Phe	7.33 (m)	3.71 ± 1.87 [a,b,c]	7.5 x 10^{-5}	–	–	–	–
Ser	3.97 (dd)	3.34 ± 1.75 [a]	1.7 x 10^{-4}	2.45 ± 1.50	7.0 x 10^{-3}	1.82 ± 1.34	9.5 x 10^{-3}
Tau	3.42 (t)	4.74 ± 2.21 [a]	9.4 x 10^{-6}	3.35 ± 1.75 [a]	1.8 x 10^{-4}	–	–
Tyr	6.91 (d)	4.69 ± 2.19 [a,b,c]	1.0 x 10^{-5}	–	–	–	–
Val	1.05 (d)	5.15 ± 2.35 [a,b,c]	4.5 x 10^{-6}	–	–	–	–
Choline compounds							
Choline	3.21 (s)	6.87 ± 2.97 [a]	2.8 x 10^{-7}	2.68 ± 1.56 [a]	2.7 x 10^{-3}	2.85 ± 1.61 [a]	5.8 x 10^{-4}
GPC	3.24 (s)	10.06 ± 4.18 [a]	8.2 x 10^{-9}	4.01 ± 1.96 [a]	8.9 x 10^{-4}	3.06 ± 1.67 [a]	3.4 x 10^{-4}
PC	3.22 (s)	–	–	–	–	2.23 ± 1.44 [a]	2.3 x 10^{-3}
Nucleotides and derivatives							
Ado/Ino	4.28 (q)	–	–	-1.56 ± 1.29	2.2 x 10^{-2}	–	–
ATP	4.39 (m)	–	–	-2.76 ± 1.58 [a]	7.4 x 10^{-4}	–	–
GTP	5.94 (d)	-2.91 ± 1.62 [a,b]	5.0 x 10^{-4}	-2.62 ± 1.54 [a]	1.1 x 10^{-3}	–	–
NAD^+	8.43 (s)	–	–	-3.71 ± 1.86 [a]	7.5 x 10^{-5}	-3.65 ± 1.85 [a]	8.7 x 10^{-5}
UDP-GlcA	7.96 (d)	-2.20 ± 1.43 [a]	2.8 x 10^{-3}	–	–	–	–
Uridine	7.87 (d)	–	–	4.28 ± 2.05 [a,c]	2.3 x 10^{-5}	–	–
UXP	5.99 (m)	-2.22 ± 1.44	2.5 x 10^{-2}	-4.76 ± 2.21 [a]	8.9 x 10^{-6}	–	–
Organic acids							
Acetate	1.92 (s)	2.67 ± 1.56 [a]	9.9 x 10^{-4}	1.98 ± 1.38	6.4 x 10^{-3}	–	–
Formate	6.52 (s)	2.06 ± 1.40	5.2 x 10^{-3}	1.95 ± 1.38	7.1 x 10^{-3}	2.01 ± 1.39	6.0 x 10^{-3}
Fumarate	1.33 (d)	–	–	-2.10 ± 1.41 [a]	4.5 x 10^{-3}	–	–
Succinate	2.40 (s)	-2.87 ± 1.61 [a]	5.8 x 10^{-4}	–	–	–	–
Other compounds							
2-HBA	0.89 (t)	2.79 ± 1.59 [a,b,c]	7.0 x 10^{-4}	4.83 ± 2.24 [a]	9.0 x 10^{-5}	1.85 ± 1.35	6.5 x 10^{-3}
β-Glucose	5.24 (d)	7.03 ± 3.03 [a]	2.6 x 10^{-7}	–	–	–	–
Cadaverine	3.02 (t)	2.46 ± 1.50 [a,b,c]	1.7 x 10^{-3}	1.84 ± 1.35 [a,c]	9.6 x 10^{-3}	1.90 ± 1.36	8.2 x 10^{-3}
Creatine	3.04 (s)	-5.66 ± 2.53 [a]	1.9 x 10^{-6}	-4.06 ± 1.98 [a,b,c]	3.6 x 10^{-5}	-2.51 ± 1.51 [a]	1.4 x 10^{-3}

DMA	2.73 (s)	4.11 ± 2.00 [a,b,c]	3.9 x 10^{-5}	2.13 ± 1.42 [a]	4.1 x 10^{-3}	2.09 ± 1.41 [a,c]	4.7 x 10^{-3}
Ethanol	1.18 (t)	–	–	2.30 ± 1.46 [a]	9.3 x 10^{-3}	2.65 ± 1.55 [a,e]	1.0 x 10^{-3}
GSH	3.79 (m)	3.66 ± 1.85 [a]	8.4 x 10^{-5}	4.64 ± 2.17 [a]	1.7 x 10^{-5}	–	–
Hypoxanthine	8.20 (s)	–	–	-2.94 ± 1.63 [a,b,c]	4.7 x 10^{-4}	-3.02 ± 1.66 [a,b]	3.8 x 10^{-4}
m-Inositol	4.06 (t)	-1.82 ± 1.35 [b,c]	9.5 x 10^{-3}	-4.23 ± 2.04 [a]	2.5 x 10^{-5}	-2.34 ± 1.47 [a]	2.0 x 10^{-3}

[a] Metabolite variation remaining significant after Bonferroni correction [57]; newly identified variations compared to [b] cDDP-treated MG-63 and [c] OXA-treated MG-63 cells.

Table S5. Metabolite variations in MG-63 cells exposed to 24 µM Pd_2Spm at 24, 48, and 72 h, compared to controls. Only statistically significant differences are shown (*p*-value < 0.05), also compliant with effect size modulus ∣ES∣ > 0.50, ES error < 75%. 3-Letter codes are used for amino acids; other abbreviations as defined in Table S1.

**Metabolite**	**δH ppm (multiplicity)**	**Pd_2Spm**					
		**24 h**		**48 h**		**72h**	
		**ES ± Error**	***p*-value**	**ES ± Error**	***p*-value**	**ES ± Error**	***p*-value**
Amino acids							
Ala	1.48 (d)	–	–	-2.80 ± 1.59 [a,d]	6.8 x 10^{-4}	–	–
Gln	2.47 (m)	-2.36 ± 1.47 [a]	2.2 x 10^{-3}	–	–	–	–
Gly	3.56 (s)	-1.89 ± 1.36	8.3 x 10^{-3}	-2.16 ± 1.42 [a,b,d]	3.8 x 10^{-3}	-5.24 ± 2.38 [a]	3.6 x 10^{-6}
His	7.08 (s)	3.10 ± 1.68 [a]	3.3 x 10^{-4}	–	–	–	–
Ile	0.94 (t)	2.85 ± 1.61 [a,b]	5.8 x 10^{-4}	–	–	–	–
Leu	0.96 (t)	2.36 ± 1.47 [a]	2.2 x 10^{-3}	–	–	–	–
Phe	7.33 (m)	2.36 ± 1.47 [a]	2.2 x 10^{-3}	–	–	–	–
Ser	3.97 (dd)	2.88 ± 1.61 [a]	5.5 x 10^{-4}	–	–	2.78 ± 1.59 [a]	7.1 x 10^{-4}
Tau	3.42 (t)	–	–	2.35 ± 1.47 [b]	8.2 x 10^{-3}	–	–
Val	1.05 (d)	2.61 ± 1.54 [a,b]	1.2 x 10^{-3}	–	–	–	–
Choline compounds							
Choline	3.21 (s)	4.84 ± 2.24 [a,c]	7.7 x 10^{-6}	4.03 ± 1.97 [a]	3.8 x 10^{-5}	2.09 ± 1.41 [a]	4.7 x 10^{-3}
GPC	3.24 (s)	1.86 ± 1.35 [c]	9.3 x 10^{-3}	4.04 ± 1.97 [a,c]	3.8 x 10^{-5}	3.30 ± 1.74 [a]	1.2 x 10^{-3}
PC	3.22 (s)	1.88 ± 1.36	8.5 x 10^{-3}	19.17 ± 7.75 [a,b]	1.5 x 10^{-11}	6.80 ± 2.95 [a]	3.1 x 10^{-7}
Nucleotides and derivatives							
Ado/Ino	4.28 (q)	–	–	2.77 ± 1.58 [a,b]	7.2 x 10^{-4}	–	–
GTP	5.94 (d)	–	–	-2.94 ± 1.63 [a]	4.7 x 10^{-4}	–	–
UDP-GlcA	7.96 (d)	2.78 ± 1.58 [a,d]	7.1 x 10^{-4}	3.02 ± 1.66 [a,d]	3.8 x 10^{-4}	–	–
Uridine	7.87 (d)	–	–	3.48 ± 1.80 [a]	1.3 x 10^{-4}	2.57 ± 1.53 [a,b,d]	1.2 x 10^{-3}
Organic acids							
Acetate	1.92 (s)	3.40 ± 1.77 [a]	1.0 x 10^{-4}	6.76 ± 2.93 [a]	3.7 x 10^{-7}	3.12 ± 1.68 [a]	3.2 x 10^{-4}
Formate	6.52 (s)	2.59 ± 1.53 [a]	2.0 x 10^{-3}	5.19 ± 2.36 [a]	4.2 x 10^{-6}	2.61 ± 1.54 [a]	1.2 x 10^{-3}
Fumarate	1.33 (d)	-3.49 ± 1.80 [a,d]	9.9 x 10^{-5}	–	–	–	–
Lactate	2.40 (s)	–	–	-2.68 ± 1.56 [a,d]	9.2 x 10^{-4}	-2.07 ± 1.40 [d]	5.0 x 10^{-3}
Succinate	1.92 (s)	-2.39 ± 1.48	6.5 x 10^{-3}	–	–	–	–
Other compounds							
2-HBA	0.89 (t)	2.94 ± 1.63 [a]	4.7 x 10^{-4}	3.26 ± 1.73 [a]	2.1 x 10^{-4}	–	–
Cadaverine	3.02 (t)	–	–	5.16 ± 2.35 [a]	4.4 x 10^{-6}	2.28 ± 1.45 [a]	2.7 x 10^{-3}
Creatine	3.04 (s)	-4.80 ± 2.23 [a]	8.4 x 10^{-6}	2.29 ± 1.46 [a]	2.6 x 10^{-3}	–	–
DMA	2.73 (s)	–	–	1.92 ± 1.37	7.7 x 10^{-3}	–	–
GSH	3.79 (m)	1.90 ± 1.36 [b]	8.2 x 10^{-3}	6.74 ± 2.93 [a,b]	3.8 x 10^{-7}	2.05 ± 1.40 [d]	5.6 x 10^{-3}
m-Inositol	4.06 (t)	–	–	-3.87 ± 1.92 [a,b]	6.0 x 10^{-5}	-2.02 ± 1.39	5.8 x 10^{-3}

[a] Metabolite variation remaining significant after Bonferroni correction [57]; [b] metabolite variations reported previously for Pd_2Spm-treated MG- 63 cells [21]; [c] metabolite variations noted in different direction compared to previous report for Pd_2Spm-treated MG-63 cells [21]; [d] newly identified variations compared to Pt_2Spm-treated MG-63 cells.

Figure S1

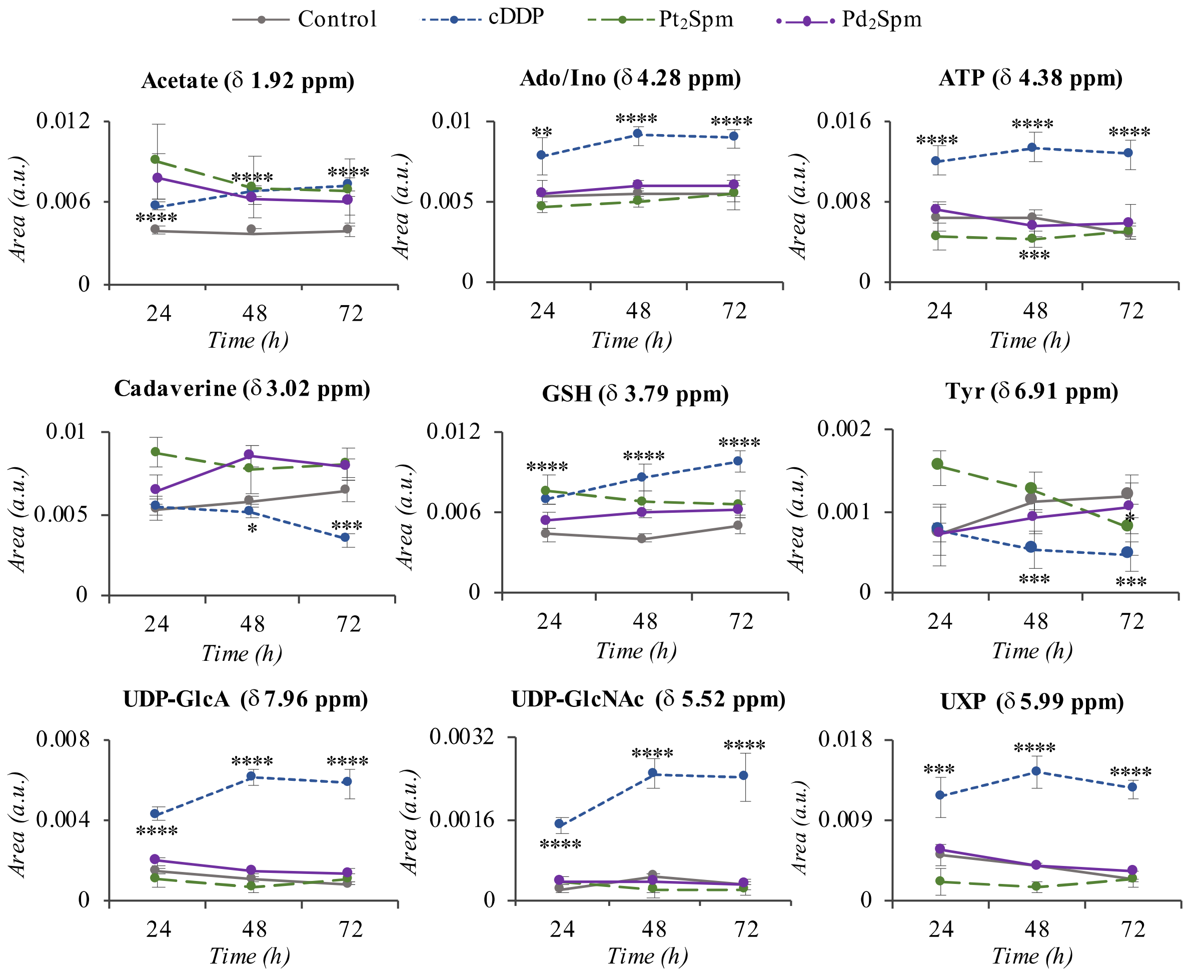

Figure S1. Time course changes for selected metabolites in MG-63 cells exposed to Pt2Spm, compared to cDDP and controls. Graphs represent peak areas normalized by total spectral area, as a function of time. Control conditions (grey solid line), exposure to 30 µM cDDP (blue dotted line), 240 µM Pt2Spm (green long dashed line) or 24 µM Pd2Spm (purple twodashed line). * p-value < 0.05; ** p-value < 0.01; *** p-value < 0.001; **** p-value < 0.0001; only indicated for cDDP for the sake of graph clarity; the reader is directed to Tables S2-S5 or Figure 4 to consult statistical significance for each metabolite variation. Abbreviations: 3-letter code used for amino acids, ATP: adenosine triphosphate; GSH: glutathione (reduced); UDP-GlcA: uridine 5′-diphosphoglucuronic acid; UDP-GlcNAc: uridine 5′-diphospho-N-acetylglucosamine; UXP: either of UDP/UTP/UDP-Gal/GlcNAc species.
